# Sociodemographic differences in 24-hour time-use behaviours in New Zealand children

**DOI:** 10.1186/s12966-022-01358-1

**Published:** 2022-10-04

**Authors:** Leila Hedayatrad, Tom Stewart, Sarah-Jane Paine, Emma Marks, Caroline Walker, Scott Duncan

**Affiliations:** 1 School of Sport and Recreation, Department of Behavioural Nutrition and Physical Activity, Auckland University of Technology, Auckland, New Zealand; 2 Te Kupenga Hauora Māori, University of Auckland, Auckland, New Zealand; 3 Growing Up in New Zealand, School of Population Health, University of Auckland, Auckland, New Zealand

**Keywords:** Time-use behaviours, Physical activity, Sedentary behaviour, Sleep, Screen time, Compositional data analysis

## Abstract

**Background:**

The time that children spend in physical activity, sedentary behaviour, and sleep each day (i.e., 24-h time-use behaviours), is related to physical and mental health outcomes. Currently, there is no comprehensive evidence on New Zealand school-aged children’s 24-h time-use behaviours, adherence to the New Zealand 24-h Movement Guidelines, and how these vary among different sociodemographic groups.

**Methods:**

This study utilises data from the 8-year wave of the *Growing Up in New Zealand* longitudinal study. Using two Axivity AX3 accelerometers, children’s 24-h time-use behaviours were described from two perspectives: activity intensity and activity type. Compositional data analysis techniques were used to explore the differences in 24-h time-use compositions across various sociodemographic groups.

**Results:**

Children spent on average, 31.1%, 22.3%, 6.8%, and 39.8% of their time in sedentary, light physical activity, moderate-to-vigorous physical activity, and sleep, respectively. However, the daily distribution of time in different activity types was 33.2% sitting, 10.8% standing, 7.3% walking, 0.4% running, and 48.2% lying. Both the activity intensity and activity type compositions varied across groups of child ethnicity, gender, and household income or deprivation.

The proportion of children meeting each of the guidelines was 90% for physical activity, 62.5% for sleep, 16% for screen time, and 10.6% for the combined guidelines. Both gender and residence location (i.e., urban vs. rural) were associated with meeting the physical activity guideline, whereas child ethnicity, mother’s education and residence location were associated with meeting the screen time guideline. Child ethnicity and mother’s education were also significantly associated with the adherence to the combined 24-h Movement Guidelines.

**Conclusions:**

This study provided comprehensive evidence on how New Zealand children engage in 24-h time-use behaviours, adherence to the New Zealand 24-h Movement Guidelines, and how these behaviours differ across key sociodemographic groups. These findings should be considered in designing future interventions for promoting healthy time-use patterns in New Zealand children.

**Supplementary Information:**

The online version contains supplementary material available at 10.1186/s12966-022-01358-1.

## Background

There has been growing evidence that time-use behaviours comprised of physical activity [1], sedentary behaviour (including screen-based activities) [2] and sleep [3] are linked with physical and mental health outcomes in school-aged children and youth. However, most of this evidence is based on studies examining the time spent in each behaviour in isolation, ignoring the intrinsic interplay between them [4]. Since daily time-use behaviours are bounded to 24-h per day, the time spent in one behaviour is co-dependent on the remaining behaviour(s) [4]. That is, an increase in the time allocated to one behaviour (e.g., physical activity) leads to less time for the remaining behaviours (e.g., sedentary behaviour and/or sleep). Favourable health outcomes (e.g., decreased body mass index) might not be merely due to the increase in one activity (e.g., physical activity), but changes in the remaining activities (less sedentary and/or more sleep). To fully understand the relationships between health and time-use behaviours, researchers are moving away from investigating these behaviours as independent correlates of health, and towards an integrated approach exploring the associations between compositions of behaviours and health [5]. This integrated approach has been conceptualised in a newly established health research area called time-use epidemiology [6].

Advocating this approach, Canada pioneered the 24-h Movement Guidelines for children and youth, which integrated the previous distinct guidelines for each behaviour [7]. This has been followed by other countries including New Zealand [8]. These guidelines contain integrated recommendations on daily amounts of moderate-to-vigorous physical activity (MVPA) (at least 60 minutes), screen time (not more than 2 h), and sleep (9–11 h for 5–13 year old children and 8–10 h for those aged 14–17 years old) for optimal health and wellbeing in children aged 5–17 years old [8]. Meeting these guidelines have been associated with favourable health indicators in children [5], and yet international studies suggest that only a small proportion of children regularly meet all these recommendations. For example, findings from a 12-country study suggest that only 7% of children aged 9–11 years met all three guidelines [9]. Findings from limited studies suggest that sociodemographic factors and parental factors (age and education) were associated with adherence to these guidelines [10, 11]. These findings on the potential sociodemographic correlates of the time-use behaviours can help to tailor future interventions aimed at promoting optimal time-use patterns. Currently, there is no comprehensive evidence on the prevalence of meeting these guidelines among New Zealand school-aged children and the associated sociodemographic factors.

To date, accelerometers have been commonly used to derive the daily activity compositions in 24-h time-use research, with the majority of the studies focusing on quantifying the time spent in different activity intensities (e.g. sedentary, light-intensity physical activity (LPA), MVPA) using count-based methods [12–15]. Here, the acceleration data are converted into “activity counts” using proprietary algorithms [16]. Subsequently, cut-points are applied to these counts to distinguish between different activity intensities. There are various and often conflicting sets of cut-points to estimate the amount of MVPA, LPA and sedentary time, reducing the comparability across studies [17]. To help address this issue, extracting activity type and posture from raw accelerometer data through machine learning and other algorithms is gaining interest [18]. These techniques are capable of detecting postures (i.e., sitting, standing, lying) and other ambulatory activities (i.e. walking and running) with high accuracy in both lab and free-living settings [19, 20]. However, to the best of our knowledge, no study has yet quantified complete 24-h time-use behaviours in terms of activity type in children (i.e., sitting, standing, walking, running, lying).

Activity researchers are increasingly using compositional data analysis (CoDA) to analyse 24-h time-use behaviours to adequately account for the compositional properties of time-use data [21]. Using this statistical approach, the aims of this study were 1) to describe the 24-h time-use behaviours of New Zealand children, both in terms of activity intensity and activity type, 2) to examine differences in 24-h time-use behaviours among different sociodemographic groups, and 3) to determine the adherence to the individual and combined 24-h Movement Guidelines for New Zealand children.

## Methods

### Data source and study participants

This study is a secondary analysis of children participating in the 8-year data collection wave (when the children were 8 years old) of the *Growing Up in New Zealand* study (GUiNZ) – an ongoing longitudinal cohort study which started in 2009. In the GUiNZ study, data have been collected at several time points, although the current study makes use of the 8-year dataset, and several sociodemographic variables collected at birth (antenatal dataset). Additional details regarding this study are available elsewhere [22]. A total of 5556 children participated in the 8-year wave of this study; however, accelerometers were only worn by a subsample of children. In total, 952 children wore accelerometers, although the final analytic sample used in this study was 623 children.

### Measurements

#### 24-hour time-use behaviours

The Axivity AX3 accelerometer was used to assess the 24-h time-use behaviours. A pair of Axivity AX3 accelerometers were placed on the dominant thigh and lower back using medical dressing or purpose-built foam pouches [23]. Participants were asked to wear these monitors for seven consecutive days. The devices were initialised to collect data at a sampling rate of 100 Hz and were downloaded using the Open Movement Software (OMGUI, version 1.0.0.30, open Movement, Newcastle University, UK). Wear/non-wear time was detected using the in-built temperature sensor following procedures described elsewhere [23, 24]. Children needed to have valid accelerometer data (both thigh and lower back) for at least 1 day over the 7 days of measurement to be included in the study. A valid day was defined as 24-h of concurrent wear time for both sensors. Two separate 24-h time-use compositions were created, one for activity intensity (based on energy expenditure) and one for activity type (based on posture). For the activity intensity composition, raw data from the back sensor were converted into counts that are congruent with the ActiGraph GT3X + device, using published algorithms [25, 26]. The scaled Evenson cut-points were then applied to categorise each 5-second epoch as sedentary, LPA or MVPA [27]. Sleep duration was derived using the Tudor-Locke algorithm for the centre of mass [28]. The minutes of each behaviour per day were averaged over the number of valid days for each participant. For the activity type composition, machine learning models were applied to each 5-second epoch from the thigh and lower back monitors. These models were previously developed and tested in both lab and free-living settings in children [19, 20]. The activity intensity composition was comprised of the following four parts: sedentary, LPA, MVPA, and sleep. For activity type, a 5-part composition was created containing: sitting, standing, walking, running, and lying.

#### 24- hour Movement Guidelines adherence

Using the 24-h Movement Guidelines for New Zealand children, participants were classified as meeting the MVPA guideline if they had accumulated on average 60+ minutes of MVPA daily. To assess adherence to the screen time guideline, each child’s mother was asked to report the hours and minutes that their child usually 1) watched television including free-to-air, online, and pay-tv or DVDs, either on TV or other screen-based devices, 2) spent time doing activities or tasks, e.g. homework, playing games, or sending messages, on any screen-based devices including computers, laptops, tablets, smartphones or gaming devices, separately for a weekday and weekend day. The responses to these two questions were summed to calculate total screen time (for the weekday and weekend day separately). Subsequently, the average of these values was calculated to obtain average daily screen time. Children who engaged in less than 2 h of screen time per day were categorised as meeting the screen time recommendation. Finally, children with 9–11 h of sleep per 24-h were categorised as meeting the sleep guideline.

#### Sociodemographic variables

Child’s age at the time of data collection was calculated using their date of birth, and mother’s age was calculated at the date of delivery (i.e., age of the mother when the child was born). The child ethnicity was classified into the following major ethnic groups: 1) European, 2) Māori, 3) Pacific 4) Asian, 5) Middle Eastern, Latin American, and African (MELAA), and 6) Other. MELAA and Other were combined as “Other” due to small numbers in each group. Children who answered, “I don’t know” to the ethnicity question were also categorised as “Other”. Household annual income and New Zealand Deprivation index 2013 (NZDep2013) [29] were used as proxy for household socioeconomic status. Mothers were asked to report their household income over the past 12 months, which was categorised into four groups: (NZD < $70,000, 70,000–100,000, 100,000–150,000, and > 150,000). NZDep2013 reflects the area-level deprivation status for each meshblock (small geographic census unit) based on nine variables from the 2013 census data. Each meshblock is assigned a deprivation score ranging from decile 1 (least deprived) to decile10 (most deprived). From these scores three categories were created: 1) low deprivation (deciles 1–3), 2) medium deprivation (deciles 4–7), and 3) high deprivation (deciles 8–10). Residence location was categorised as either urban or rural.

Information on the mother’s highest level of education was obtained from the antenatal dataset (as it is not available in the 8-year dataset). Mothers were asked about their highest qualification at the time and could choose from the following categories: 1) without a secondary school qualification, 2) secondary school/National Certificate of Educational Achievement (NCEA) levels 1–4, 3) diploma/Trade certificate/NCEA levels 5–6, 4) bachelor’s degree 5) higher degree. These categories were dichotomised into 1) “less than a bachelor's degree” and 2) “bachelor’s degree or higher”. Mother’s weekly work hours were obtained from the 8-year datasets and subsequently categorised as < 15, 15–30, 30–40 and ≥ 40 hours. Information on family structure was categorised as either: 1) single parent 2) both parents 3) parent(s) with extended family or parent(s) living with non-kin.

### Statistical analysis

All the analyses were carried out in R (version 3.6.1; The R Foundation for Statistical Computing, Vienna, Austria). Descriptive characteristics including frequency (categorical variables) and means (continuous variables) were calculated. Sociodemographic differences between children with and without accelerometer data at the 8-year time point were compared using Chi-squared tests and independent samples t-tests for continues variables. This study used a compositional data analysis approach. Firstly, missing values and parts of each composition that contained zeros were imputed using log-ratio expectation-maximisation [30]. This method of zero imputation has been shown to produce the least bias [31]. For descriptive statistics, the geometric mean was calculated for time spent in each activity intensity and activity type component, and then normalised to 1440 minutes (24-h) to obtain the compositional mean for each activity over a 24-h period. The variation array was used to describe the variability within each composition.

Compositional multivariate analysis of variance (MANOVA) was used to compare activity intensity and activity type components among different sociodemographic groups (i.e., gender, ethnicity, mother’s age, mother’s education, mother’s work hours, household structure, household income, household deprivation, and residence location) [32]. Compositional parts were first transformed using the isometric log-ratio transformation, before being entered into each model as the dependant variable. For each model, partial eta squared (η_p_^2^) was calculated as an indication of effect size. Hoteling’s T-square tests with the Holm adjustment were applied as post-hoc comparisons for sociodemographic variables with more than two levels [32]. To identify the specific component(s) of each composition responsible for significant overall differences, between-group log-ratio differences along with bootstrapped 95% confidence intervals were estimated for each component [32]. These estimated log-ratio differences were back-transformed into percentages using the following formula: (exp (log-ratio difference) – 1) * 100. These differences were also visualised using compositional geometric mean bar plots and ternary plots.

Lastly, the association between meeting each component of the 24-h Movement Guidelines and sociodemographic factors was assessed using Chi-squared tests. In cases where the expected cell counts were less than 5 (six occurrences), the Fisher’s exact test was used instead. For each test, Cramer’s V was calculated as an indication of effect size. Statistical significance was set at 0.05 for all analyses.

## Results

Of the 5,556 participants in the 8-year wave of GUiNZ, 623 (51.5% girls, mean age = 7.8 (0.24) years old) had valid accelerometer data for at least one complete day (24 h wear time) for both sensors simultaneously, making them eligible for this study. Other reasons for exclusion were: only had data from one sensor (*n* = 79; either due to the child only consenting to wearing one sensor, a lost sensor, or data were unable to be downloaded), corrupt raw data or failure to time synchronise the thigh and back sensors (*n* = 30). The mean number of valid days was 4.9, which contained, on average, 1.3 weekend days.

Table 1 describes the characteristics of the GUiNZ participants with and without accelerometer data. Sociodemographic characteristics varied between those with and without accelerometer data in terms of child age, ethnicity, mother’s age, mother’s education, mother’s working hours, household income, and household deprivation. No significant differences were seen for gender, residence location, or household structure.

**Table 1:** Characteristics of the participants in the 8-year wave of the GUiNZ (with/without accelerometer data)

Variable	Participants without accelerometer data n (%) or mean ***n*** = 4856	Participants with accelerometer data n (%) or mean ***n*** = 623	***p*** value^*^
**Age (years)**	7.6 (0.26)	7.8 (0.24)	**< 0.001**
**Gender**			0.117
Boy	2516 (51.8)	302 (48.5)	
Girl	2340 (48.2)	321 (51.5)	
**Ethnicity**			**0.003**
European	1623 (38.2)	273 (44.2)	
Māori	968 (22.8)	119 (19.3)	
Pacific	470 (11.1)	45 (7.3)	
Asian	460 (10.8)	74 (12.0)	
Other	727 (17.1)	107 (17.3)	
Missing	608	< 10	
**Mother’s age at delivery (years)**			**< 0.001**
≤ 20	276 (5.7)	13 (2.1)	
≤ 25	720 (14.8)	66 (10.6)	
≤ 30	1284 (26.4)	155 (24.9)	
≤ 35	1585 (32.6)	237 (38.0)	
≤ 40	864 (17.8)	137 (22.0)	
> 40	126 (2.6)	15 (2.4)	
Missing	< 10	0	
**Mother’s level of education**			**< 0.001**
Less than a bachelor’s degree	2804 (63.0)	300 (48.2)	
Bachelor’s degree or higher	2039 (37.0)	323 (51.8)	
Missing	13	0	
**Mother’s work hours**			**0.001**
< 15	1807 (41.2)	181 (31.5)	
15–30	819 (18.7)	121 (21.0)	
30–40	731(16.7)	107 (18.6)	
≥ 40	1030 (23.4)	166 (28.9)	
Missing	469	48	
**Household structure**			0.176
Single parent	433 (9.5)	62 (10.0)	
Both parents	3172 (69.5)	447 (72.2)	
Parent(s) with extended family or non-kin	958 (21.0)	110 (17.8)	
Missing	293	< 10	
**Household income**			**0.001**
≤ 70 k	1092 (29.9)	118 (21.0)	
70–100 k	655 (18.0)	120 (21.4)	
100–150 k	860 (23.6)	141 (25.1)	
> 150 k	1040 (28.5)	183 (32.6)	
Missing	1209	61	
**Household deprivation**^a^			**< 0.001**
Low (1–3)	1576 (35.1)	222 (35.8)	
Medium (4–7)	1639 (36.5)	276 (44.5)	
High (8–10)	1280 (28.5)	122 (19.7)	
Missing	361	< 10	
**Residence location**			0.350
Urban	3973 (88.4)	540 (87.1)	
Rural	522 (11.6)	80 (12.9)	
Missing	361	< 10	
**Screen time (min)**	278	272	0.607
Missing	1949	151	

^*^Chi-square test (categorical variables) or Independent sample t-test (continuous variables)

^a^According to the New Zealand Index of Deprivation 2013

Of the 623 children with valid accelerometer data, information on activity intensity and activity type compositions could be extracted for 620 and 602 children, respectively (due to algorithm or imputation errors). Table 2 shows the compositional means of time spent in each component of the activity intensity and activity type compositions for the total sample, and separately by gender, ethnicity, and other sociodemographic factors. From the activity intensity perspective, children spent on average, 31.1%, 22.3%, 6.8%, and 39.8% of their time in sedentary, LPA, MVPA, and sleep, respectively. However, the daily distribution of time in different activity types was 33.2% sitting, 10.8% standing, 7.3% walking, 0.4% running, and 48.2% lying down. The variation array, which illustrates the overall variation within the activity intensity and activity type compositions, are shown in Supplementary Tables S1 and S2, respectively.

**Table 2:** Compositional means (in minutes) for different components of activity intensity and activity type compositions by gender, ethnicity and sociodemographic status

	Activity intensity components (***n*** = 620)				Activity type components (***n*** = 602)				
	Sedentary	LPA	MVPA	Sleep	Sitting	Standing	Walking	Running	Lying
**Total**	448	321	98	573	479	155	106	7	694
**Gender**									
Boy	450	311	109	570	486	137	109	8	700
Girl	446	331	89	575	476	170	101	5	688
** Ethnicity**									
European	443	322	99	576	476	152	106	7	698
Māori	449	320	101	569	482	142	108	7	701
Pacific	458	307	103	572	507	141	93	5	694
Asian	458	334	91	556	496	172	100	5	666
Other	445	319	96	580	474	160	104	7	695
**Mother’s age at delivery (years)**									
≤ 20	484	294	93	569	530	132	91	4	682
≤ 25	452	321	98	570	495	151	97	6	690
≤ 30	439	328	101	571	465	160	106	7	702
≤ 35	448	325	97	571	480	154	107	7	693
≤ 40	452	312	98	578	486	148	106	7	692
> 40	454	317	85	584	511	152	89	5	683
**Mother’s education level**									
Less than a bachelor’s degree	452	319	97	571	487	153	102	6	692
Bachelor’s degree or higher	444	323	98	574	488	153	102	6	691
**Mother’s work hours**									
< 15	453	319	97	571	480	151	102	6	701
15–30	438	322	99	581	465	159	109	7	701
30–40	446	318	100	576	502	145	99	7	688
≥ 40	449	327	98	566	479	158	109	8	686
**Household structure**									
Single parent	462	309	92	576	487	141	97	6	710
Both parents	443	323	99	575	478	155	106	7	693
Parents with extended family or living with non-kin	460	321	96	563	489	151	102	6	691
**Household income**									
< 70 K	455	310	97	578	479	152	99	6	704
70–100 k	455	320	96	570	488	157	101	6	687
100–150 k	432	333	100	575	475	165	109	7	685
> 150 k	446	324	99	571	477	148	107	8	701
**Household deprivation**									
Low	449	320	97	574	476	154	108	8	694
Medium	442	324	100	574	474	155	106	7	698
High	461	320	93	566	505	149	96	5	686
**Residence location**									
Urban	451	321	97	572	484	152	104	7	694
Rural	431	326	104	578	460	167	111	7	694

*LPA* Light-intensity physical activity, *MVPA* Moderate-to-vigorous physical activity

### Activity intensity composition

**Fig. 1:**
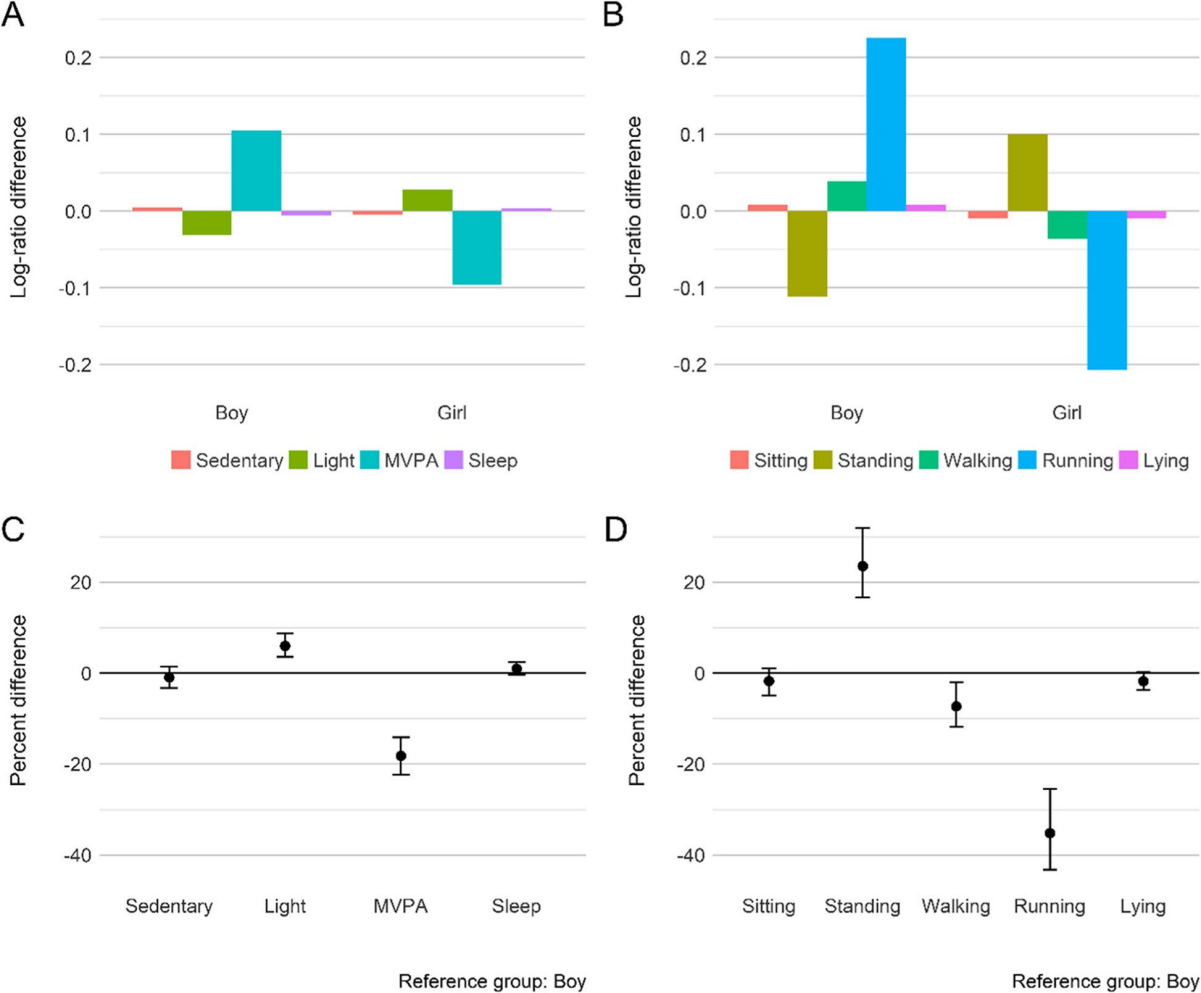
**A** and **B** Compositional geometric mean bar plots: each bar presents the log-ratio differences between the geometric mean of the entire sample and geometric mean of each activity intensity and activity type components by gender. **C** and **D** The percentage differences (with 95% confidence interval) in times spent in each activity intensity and activity type components between genders. Estimates above the reference line mean girls have a higher proportion of an activity, relative to boys (reference group). *Light* light-intensity physical activity, *MVPA* Moderate-to-vigorous physical activity

Table 3 presents the results for the MANOVA tests, which were used to compare these compositions among sociodemographic groups. For the activity intensity composition, there were significant overall differences between gender (*p* <  0.001; ηp2 = 0.19), with girls spending significantly less time in MVPA (-18%, 95% CI = -22–-14%) but more time in LPA (6%, 95% CI = 3–8%), compared to boys. However, no significant difference in sedentary and sleep time between gender was observed (Fig. 1C).

**Table 3:** Results of compositional MANOVA of differences in daily activity intensity and activity type compositions between sociodemographic factors

	Activity intensity composition					Activity type composition				
	Pillai’s trace	F	df	***p***-value	η_**p**_^**2**^	Pillai’s trace	F	df	***p***-value	η_**p**_^**2**^
**Gender**	0.187	47.26	3, 616	**< 0.001**	0.187	0.171	30.87	4, 597	**< 0.001**	0.171
**Ethnicity**	0.048	2.48	12, 1830	**0.003**	0.160	0.069	2.60	16, 2368	**< 0.001**	0.017
**Mother’s age at delivery**	0.032	1.33	15,1842	0.178	0.011	0.037	1.48	15,1788	0.106	0.012
**Mother’s education level**	0.003	0.66	3, 616	0.575	0.003	0.010	1.57	4, 597	0.179	0.010
**Mother’s work hours**	0.018	1.16	91,704	0.313	0.006	0.025	1.55	91,653	0.126	0.008
**Household structure**	0.019	2.03	6, 1224	0.057	0.009	0.014	1.07	8, 1186	0.374	0.007
**Household income**	0.031	1.96	9, 1668	**0.040**	0.010	0.039	1.79	12, 1617	**0.044**	0.013
**Household deprivation**	0.014	1.48	6, 1226	0.181	0.007	0.035	2.70	8, 1188	**0.005**	0.017
**Residence location**	0.010	2.15	3, 613	0.092	0.010	0.010	1.62	4, 594	0.167	0.010

The levels of each factor can be seen in Table 2

Bold values represent significant differences

**Fig. 2:**
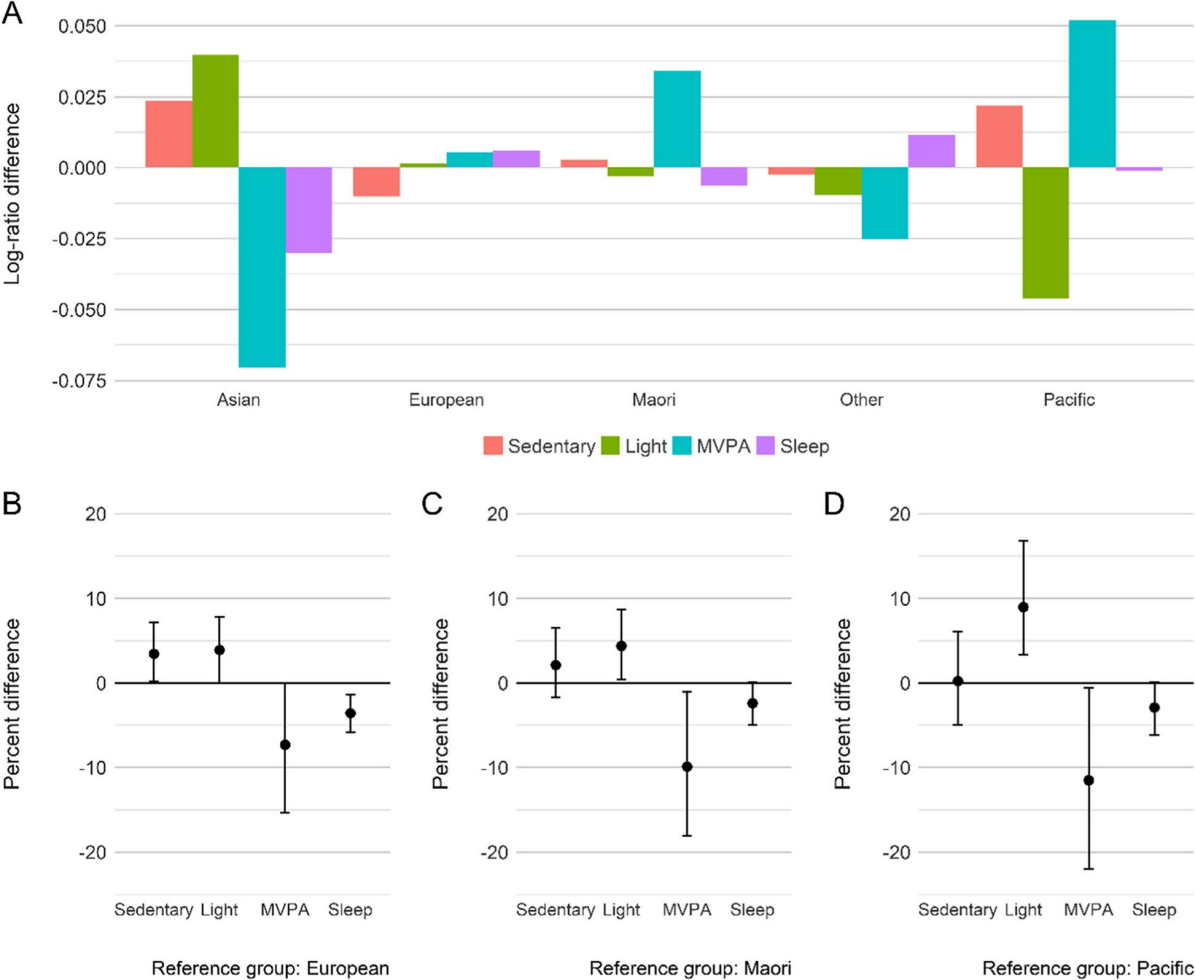
**A** Compositional geometric mean bar plots comparing the geometric mean of each activity intensity components for each child ethnicity group with the geometric mean of the entire sample. **B**, **C** and **D** The percentage differences of the geometric mean of activity intensity components between Asian and European, Asian and Māori, and Asian and Pacific, respectively. Estimates above the reference line mean that ethnic group has a higher proportion of an activity, relative to the reference group. *Light* light-intensity physical activity, *MVPA* Moderate-to-vigorous physical activity

The overall intensity composition was different among groups based on child ethnicity (*p* = 0.003; ηp2 = 0.16), and the Hoteling’s post hoc test revealed that Asian children had significantly different compositions compared to European (*p* = 0.015), Māori (*p* = 0.028) and Pacific (*p* = 0.004) children. As shown in Fig. 2, Asian children were involved in more LPA compared to European (4%, 95% CI = 0.2–7%), Māori (4%, 95% CI = 0.2–9%) and Pacific (9%, 95% CI = 3–16%) children. They also slept less than European (-3.5%, 95% CI = -6–-1%) and Pacific (-3%, 95% CI = -6 – -0.5%) children.

**Fig. 3:**
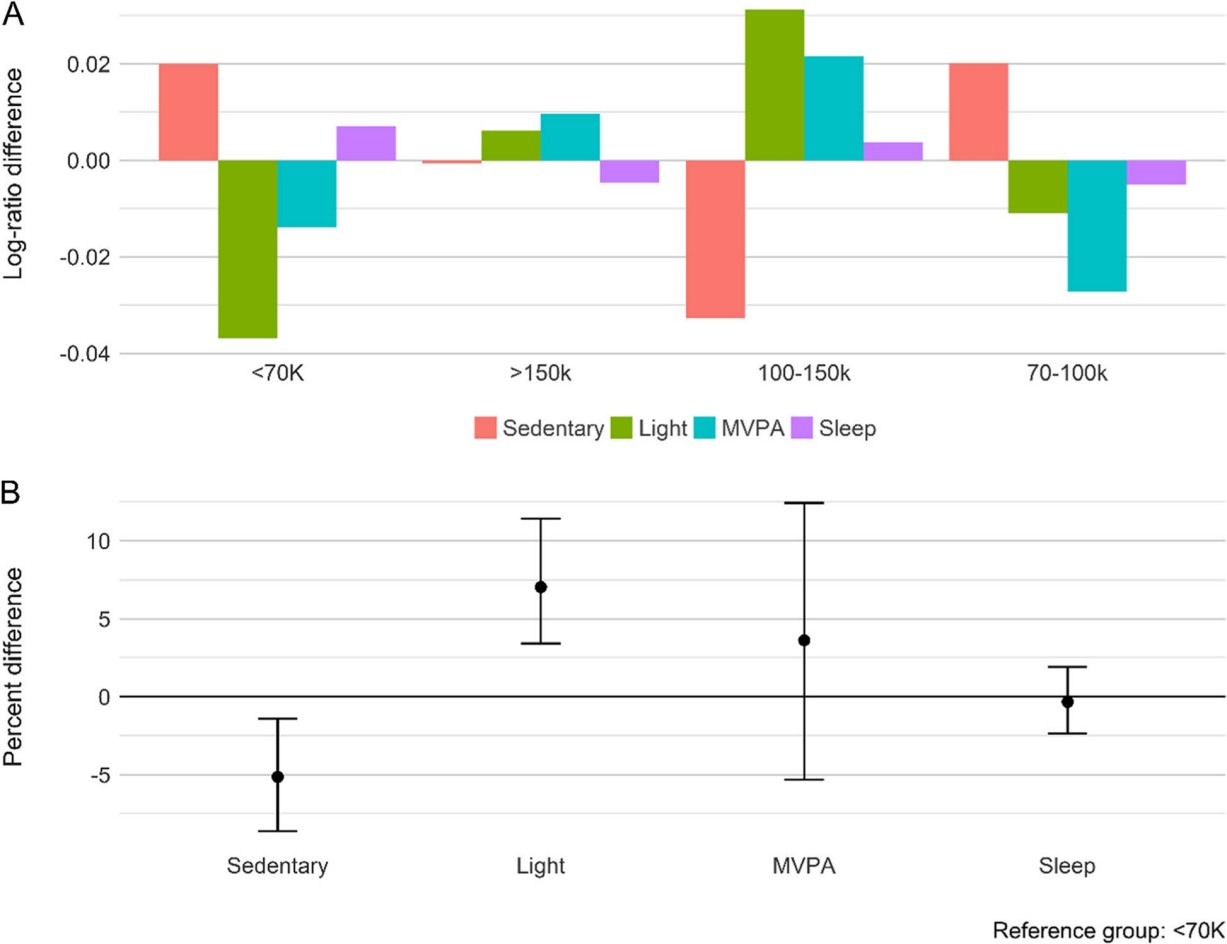
**A** Compositional geometric mean bar plots comparing the geometric mean of each activity intensity components for each household income category group with the geometric mean of the entire sample. **B** The percentage differences of the geometric mean of activity intensity components between households with less than NZ$70 K annual income and households with NZ$100–150 k annual income. Estimates above the reference line mean households with NZ$100–150 k annual income have a higher proportion of an activity, relative to < 70 k (reference group). *Light* light-intensity physical activity, *MVPA* Moderate-to-vigorous physical activity

Lastly, overall intensity compositions were different among annual household income groups (*p* = 0.04; ηp2 = 0.01), with post hoc tests revealing differences between <$70 k and $100–150 k groups (*p* = 0.022). Children from households with an annual income of $100–150 K were less sedentary (-5%, 95% CI = -8–-1%) and involved more in LPA (7%, 95% CI = 3–11%) compared to those from households with a $70 k annual income (Fig. 3).

### Activity type composition

Significant differences also existed between gender for the overall activity type composition (*p* <  0.001; ηp2 = 0.17). The percentage differences in Fig. 1D suggest that girls spent significantly more time standing (23%, 95% CI = 17–31%) and less time walking (-7%, 95% CI = -12–-1%) and running (-35%, 95% CI = -44–-25%) compared to boys.

**Fig. 4:**
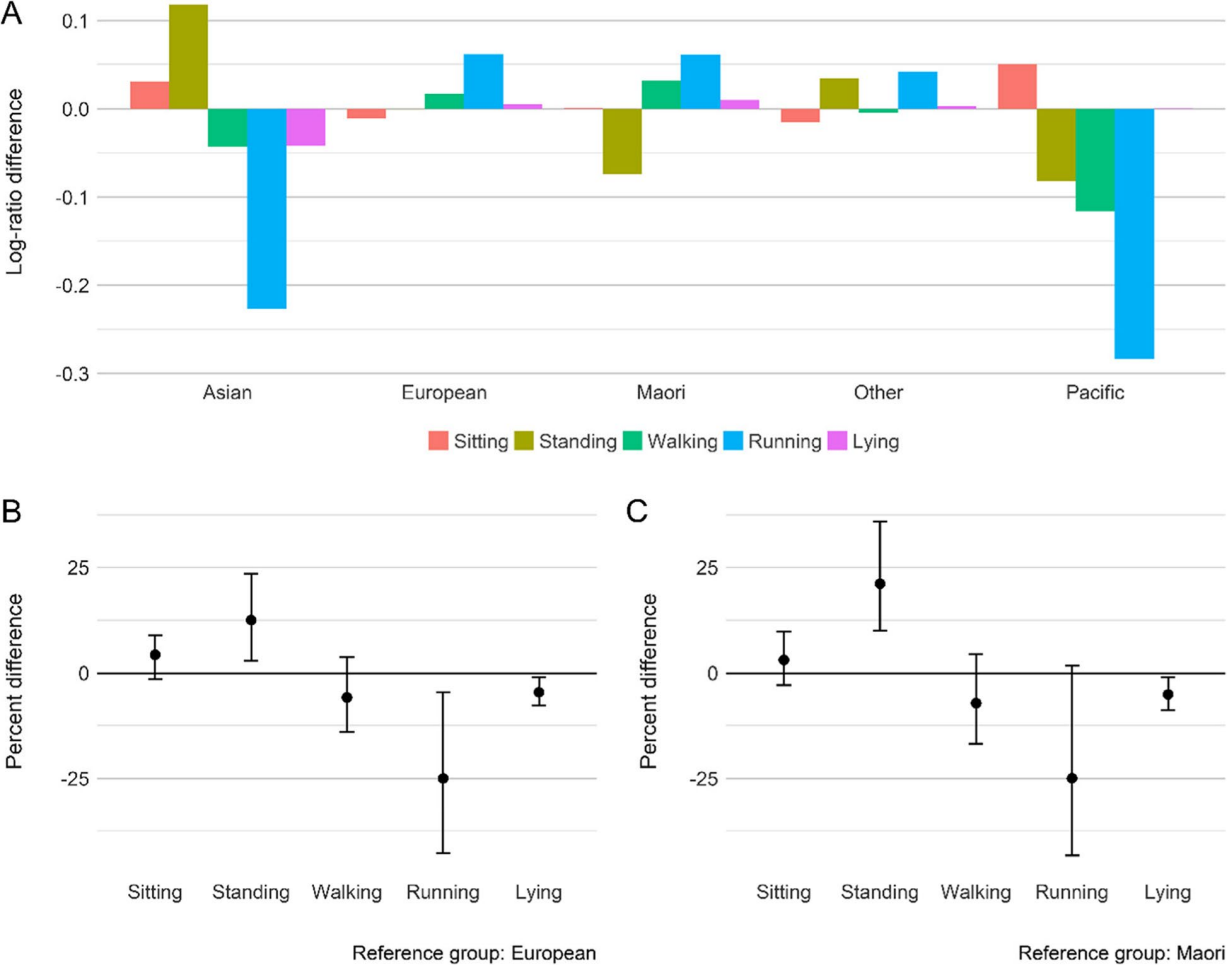
**A** Compositional geometric mean bar plots comparing the geometric mean of each activity type components for each child ethnicity group relative to the entire sample. **B** and **C** The percentage differences of the geometric mean of activity type components between Asian and European, Asian and Māori, respectively. Estimates above the reference line mean that ethnic group has a higher proportion of an activity, relative to the reference group

The overall activity type composition was different among child’s ethnicities (p <  0.001; ηp2 = 0.02), and the Hoteling’s post hoc test revealed Asian and European (*p* = 0.002), and Asian and Māori (*p* = 0.003) were different. Figure 4 shows that Asian children spent more time standing (13%, 95% CI = 3–23%) and less time running (-25%, 95% CI = -40–-3%) and lying down (-5%, 95% CI = -8 – -1%) compared to European children. Asian children also spent more time standing (21%, 95% CI = 8–34%) and less time running (-24%, 95% CI = -44–-0.3%) and lying down (-5%, 95% CI = -9–-1%) compared to Māori children.

**Fig. 5:**
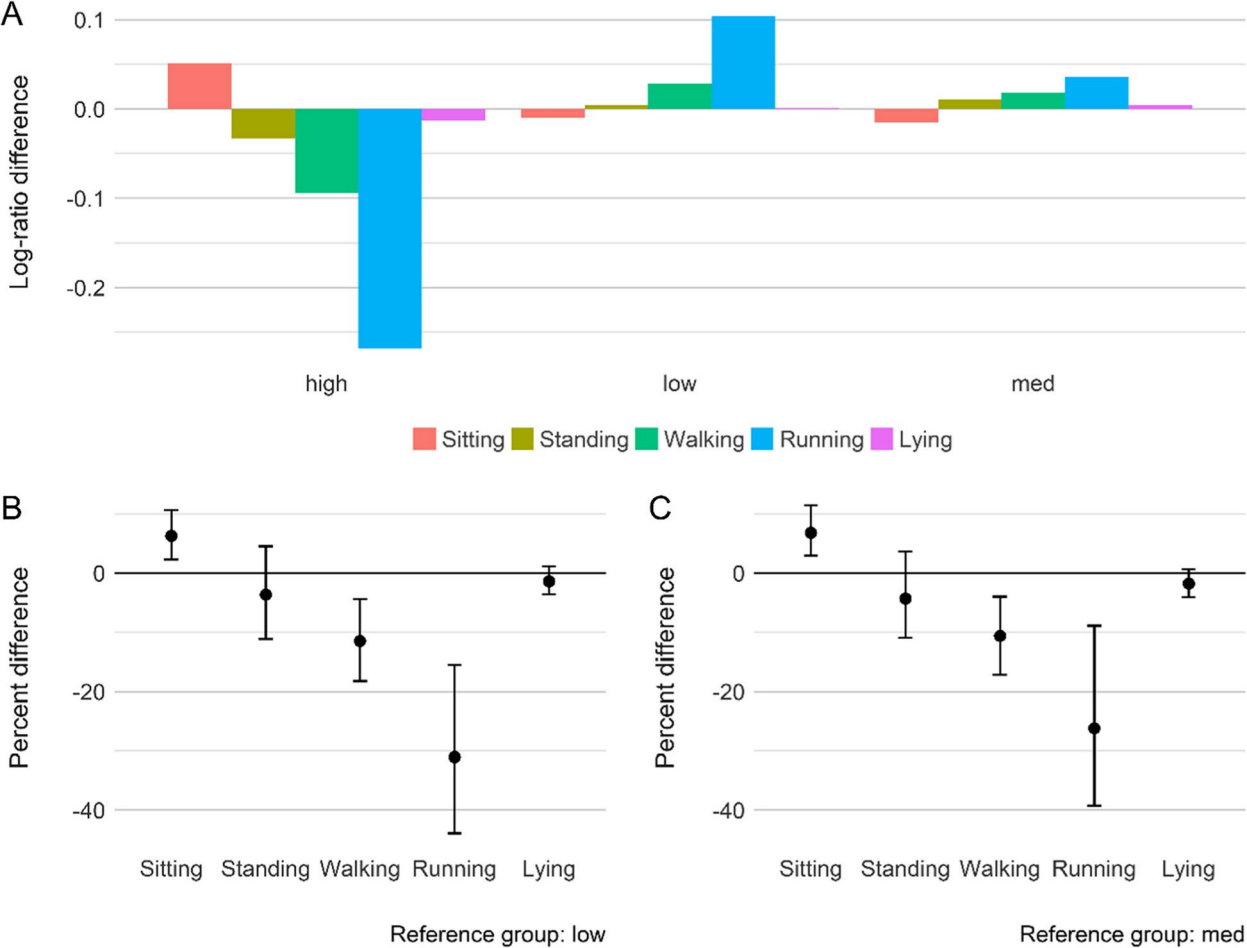
**A** Compositional geometric mean bar plots comparing the geometric mean of each activity type components for each deprivation status category relative to the entire sample. **B** and **C** The percentage differences of the geometric mean of activity type components between low and high, and medium and high levels of deprivation, respectively. Estimates above the reference line mean that level of deprivation has a higher proportion of an activity, relative to the reference group

Household deprivation was also related to the overall activity type composition (*p* = 0.005; ηp2 = 0.02); specifically, children from highly deprived areas were different from those in areas of low (*p* = 0.002) and medium (*p* = 0.012) deprivation. Children from the most deprived areas spent more time sitting (6%, 95% CI = 2–11%) and less time walking (-11%, 95% CI = -17–-3%) and running (-31%, 95% CI = -46–-16%) compared to those from areas of low deprivation. Similar contrasts were seen for medium to high deprivation (Fig. 5).

Although the MANOVA showed possible significant differences between household income groups for activity type compositions, results from the post hoc analyses did not show any significant differences between household income groups for activity type compositions after adjusting for multiple comparisons.

The intensity and activity type compositions of the children between gender, among ethnicities, and household income groups are also shown by means of ternary plots with three behaviours represented at a time (Supplementary Figures S1, S2 and S3).

### Adherence to 24-hour Movement Guidelines

Table 4 provides information on the proportion of children who met the individual and combined components of the 24-h Movement Guidelines, as well as the associated sociodemographic factors. Significantly, more boys (93.3%) than girls (86.6%) met the PA guideline (*p* = 0.006; Cramer’s V = 0.11). The percentage of children meeting the PA guideline was significantly different between children from rural and urban areas (97.5% vs. 88.6%, *p* = 0.015; V = 0.10). Meeting the screen time guideline was also associated with the child’s ethnicity (*p* = 0.021; V = 0.15), mother’s level of education (*p* = 0.006; V = 0.12 and residence location (*p* = 0.033; V = 0.10). A higher proportion of European (20.4%) and Asian children (20%), met the screen time guideline compared to Māori (7.1%) and Pacific (6.2%). Mother’s education (*p* = 0.017) and child ethnicity (*p* = 0.008; V = 0.16) were related to meeting the combined 24-h guidelines.

**Table 4:** Proportion of children meeting the MVPA, screen time, and sleep recommendations and combinations of these recommendations, and associated sociodemographic factors

	MVPA			Screen time			Sleep			MVPA+ Screen time + Sleep		
	Met	Not met	***p [V]***	***Met***	***Not met***	***p [V]***	***Met***	***Not met***	***p [V]***	***Met***	***Not met***	***p [V]***
**Total**	557 (89.5)	63 (10.2)	**–**	76(16.1)	396 (83.9)	–	387(62.4)	233 (37.6)	–	50 (10.6)	420(89.4)	–
**Gender**			**0.006** [0.11]			0.897 [0.01]			0.786 [0.01]			0.305 [0.05]
Boy	279 (93.3)	20 (6.7)		37 (15.9)	196 (84.1)		185 (61.9)	114 (38.1)		28 (12.1)	203 (87.9)	
Girl	278 (86.6)	43 (13.4)		39 (16.3)	200 (83.7)		202 (62.9)	119 (37.1)		22 (9.2)	217 (90.8)	
**Ethnicity**			0.182* [0.10]			**0.021** [0.15]			0.562 [0.07]			0.008* [0.16]
European	245 (90.1)	27 (9.9)		44 (20.4)	172 (79.6)		176 (64.7)	96 (35.3)		32 (14.9)	183 (85.1)	
Māori	112 (94.9)	< 10		< 10	78 (92.9)		68 (57.6)	50 (42.4)		< 10	80 (96.4)	
Pacific	41(91.1)	< 10		< 10	30 (93.8)		31 (68.9)	14 (31.1)		0	32 (100)	
Asian	62 (84.9)	11(15.1)		11 (20.0)	44 (80.0)		43 (58.9)	30 (41.1)		< 10	50 (90.9)	
Other	94 (87.9)	13 (12.1)					66 (61.7)	41(38.3)		10 (12.3)	71 (87.7)	
**Mother’s age at delivery (years)**			0.823* [0.06]			0.760* [0.08]			0.087 [0.12]			0.260* [0.12]
≤ 20	11 (84.6)	< 10		0	< 10		< 10	< 10		0	< 10	
≤ 25	61 (92.4)	< 10		< 10	41 (89.1)		50 (75.8)	16 (24.2)		< 10	44 (95.6)	
≤ 30	141 (91.6)	13 (8.4)		20 (16.8)	99 (83.2)		93 (60.4)	61 (39.6)		10 (8.4)	109 (91.6)	
≤ 35	209 (88.6)	27 (11.4)		29 (15.7)	156 (84.3)		136 (57.6)	100 (42.4)		21 (11.4)	163 (88.6)	
≤ 40	121 (89.0)	15 (11)		19 (18.4)	84 (81.6)		92 (67.6)	44 (32.4)		14 (13.7)	88 (86.3)	
> 40	14 (93.3)	< 10		< 10	10 (76.9)		< 10	< 10		< 10	10 (76.9)	
**Mother’s education**			0.940 [< 0.01]			**0.006** [0.12]			0.184 [0.05]			**0.017** [0.11]
less than a bachelor’s degree	268(89.9)	30 (10.1)		24 (11.1)	193 (88.9)		178 (59.7)	120 (40.3)		15 (6.9)	201 (93.1)	
Bachelor’s degree or higher	289 (89.8)	33 (10.2)		52 (20.4)	203 (79.6)		209 (64.9)	113 (35.1)		35 (13.8)	219 (86.2)	
**Mother’s work hours**			0.524 [0.06]			0.162 [0.11]			0.115 [0.10]			0.610 [0.06]
< 15	166 (92.2)	14 (7.8)		22 (17.5)	104 (82.5)		113 (62.8)	67(37.2)		14 (11.1)	112 (88.9)	
15–30	110 (90.9)	11 (9.1)		21 (21.2)	78 (78.8)		82 (67.8)	39 (32.2)		12 (12.1)	87 (87.9)	
30–40	93 (86.9)	14 (13.1)		10 (11.8)	75 (88.2)		72 (67.3)	35 (32.7)		< 10	76 (9.4)	
≥ 40	147 (89.6)	17 (10.3)		15 (11.7)	113(88.3)		91 (55.5)	73 (44.5)		< 10	117 (92.9)	
**Household structure**			0.851 [0.02]			0.217 [0.08]			0.177 [0.08]			0.156* [0.09]
Single parent	56 (91.8)	< 10		< 10	40 (88.9)		43(70.5)	18 (29.5)		< 10	41 (91.1)	
Both parents	400(89.9)	45(10.1)		61 (17.8)	282 (82.2)		280(62.9)	165 (37.1)		42 (12.3)	299(87.7)	
Parents with extended family or living with non-kin	98 (89.1)	12 (10.9)		< 10	72 (88.9)		62(56.4)	48 (43.6)		< 10	77 (95.1)	
**Household income**			0.698 [0.05]			0.442 [0.08]			0.094 [0.11]			0.519 [0.07]
< 70 K	105 (89.7)	12 (10.3)		< 10	71 (88.8)		82(70.1)	35 (29.9)		< 10	74 (93.7)	
70 –100 k	107(89.2)	13 (10.8)		13 (13.8)	81 (86.2)		65(54.2)	55 (45.8)		< 10	85 (90.4)	
100 –150 k	131(92.9)	10 (7.1)		23 (19.2)	97 (80.8)		88(62.4)	53 (37.6)		15 (12.5)	105 (87.5)	
> 150 k	163 (89.6)	19 (10.4)		25 (17.0)	122 (83.0)		112(61.5)	70 (38.5)		17 (11.6)	129 (88.4)	
**Household deprivation**			0.776 [0.03]			0.551 [0.05]			0.179 [0.07]			0.311 [0.07]
Low	201 (91.0)	20 (9.0)		25 (13.7)	157 (86.3)		134 (60.6)	87 (39.4)		14 (7.7)	167 (92.3)	
Medium	246 (89.1)	30 (10.9)		37 (17.8)	171 (82.2)		182 (65.9)	94 (34.1)		25 (12.0)	183 (88.0)	
High	107 (89.2)	13 (10.8)		13 (16.0)	68 (84.0)		68 (56.7)	52 (43.3)		10 (12.5)	70 (87.5)	
**Residence location**			**0.015** [0.10]			**0.033** [0.10]			0.198 [0.05]			0.058 [0.09]
Urban	476 (88.6)	61 (11.1)		59 (14.5)	348 (85.5)		329 (61.3)	208 (38.7)		38 (9.4)	367 (90.6)	
Rural	78 (97.5)	29 (2.5)		16 (25.0)	48 (75.0)		55 (68.8)	25 (31.2)		11 (17.2)	53 (82.8)	

*MVPA* Moderate-to-vigorous physical activity, *V* Cramer’s V effect size

^*^Fisher’s exact test

Bold values represent significant differences

## Discussion

Overall, 24-h activity intensity and activity type compositions differed by children gender, ethnicity, household income, and household deprivation. Although most children met the PA recommendation, only 62.5% and 16% of children met the sleep and screen time recommendations, respectively. The compliance to the combined 24-h Movement Guidelines was even lower (10.6%). Meeting the individual and combined 24-h Movement Guidelines was also associated with several sociodemographic factors.

### 24-hour activity intensity and activity type compositions

From an activity intensity perspective, children spent 31.1% of their day sedentary (448 minutes), 29.1% in physical activity (419 minutes; 6.8% MVPA) and 39.8% sleeping (573 minutes). These figures are comparable to the findings from a recent study in 690 New Zealand children aged 6–10 years [33]. Compared to Canadian children aged 6–17 years old, children in our study were less sedentary (~ 100 minutes less), more physically active (~ 47 more minutes in MVPA and ~ 58 minutes in LPA), and had similar amounts of sleep [34]. Similarly, children in our study were less sedentary (~ 33–125 minutes less), and more engaged in MVPA (~ 29–55 minutes more) compared 9- to 11-year-old children from 12 countries [14]. Additionally, these results suggest New Zealand children obtain almost the same amount of sleep as Australian and UK children, but more than Canadian, European, American, Asian and African children [14].

Children’s 24-h activity intensity compositions have been linked with various physical and mental health outcomes [14, 34, 35]. However, using count-based approaches to derive these activity intensity compositions is not without challenges; specifically, the detection of sedentary behaviour where all non-ambulatory activities including standing are potentially misclassified as sedentary behaviour [36]. This error in estimating sedentary behaviour may ultimately confound the true health-related impacts of the 24-h time-use compositions. On the other hand, activity type recognition models have shown high accuracy in detecting sitting, standing and other activity types [19, 20]. Using these models, we also described the 24-h activity type compositions of children, which, to our knowledge, is the first study where the 24-h compositions of children have been described from an activity type perspective.

### Sociodemographic correlates of 24-hour activity intensity and activity type compositions

In this study, boys spent significantly more time in MVPA and less time in LPA than girls, which is in accordance with previous studies identifying gender as a correlate of physical activity in children [37, 38]. In terms of activity type, girls had less walking and running time and more standing time than boys. Measuring daily activities of Malaysian children (aged 9–11 years) using activPAL, it was shown that on average girls had more standing time than boys [39], which aligns with our findings. However, in the aforementioned study, girls had significantly less lying/sitting time compared to boys during the weekend. This is distinct from our observations, where no gender differences were observed for sitting or lying time. These inconsistencies could be attributable to different accelerometers and methods for measuring daily activity types.

Differences in children’s activity intensity compositions were observed across ethnicities. Asian children spent less time in MVPA and sleep than other ethnicities, while they were engaged in more LPA and sedentary time. In Taylor et al.’s study of New Zealand children (aged 6–10 years), Asian children were less active (less MVPA and LPA) and more sedentary compared to all other ethnicities and had shorter sleep duration compared to European and Māori children [33]. This is congruent with findings from previous international studies where minoritised ethic groups had more sitting [40, 41] and lower PA [41].

Additionally, children from high-income households ($100–150 K) spent significantly more time in LPA and were less sedentary compared to children from low-income households ($70 k and less). No association was identified between the amounts of MVPA across the household income categories. In a study of Australian children (aged 9–11 years), a weak positive association was observed between household income and MVPA, but no association was observed between household income and sedentary time [42]. In that study, parental education was used jointly with household income as an indicator of social-economic status and showed no association between parental education and MVPA or sedentary time, which aligns with our findings. Contrary to these findings, a weak negative association was found between parental education and sedentary time in a study of UK school-aged children [43].

The activity intensity compositions did not differ among children from different areas of deprivation, a finding that is consistent with that reported by Taylor et al. [33]. In contrast, we found that the activity type compositions were significantly different between children from high deprivation to children from low to medium deprived households. Specifically, children from high level of deprivation had less running and walking time and more sitting time than their peers from less deprived areas. This finding highlights the importance of assessing activity type, in addition to activity intensity in order to provide a better understanding of 24-h time-use behaviours in children.

### 24-hour Movement Guidelines adherence and associated sociodemographic factors

Regarding the proportion of children meeting the individual and combined 24-h Movement Guidelines, the majority met the MVPA recommendation (90%), while 62.5% and 16% met the sleep and screen time recommendations, respectively. Significant differences were observed in meeting the MVPA guideline between genders, with higher adherence in boys as compared to girls (93.3% vs. 86.6%). This is supported by previous evidence on children from Canada [44] and Mozambique [11]. We also observed that a higher proportion of children residing in rural areas met the MVPA guideline compared to those living in urban areas (97.5% vs. 88.6%). A similar study among children (9–11 years) in Mozambique, also showed a higher prevalence of meeting the MVPA guideline among rural children [11].

Adherence to the screen time guideline was extremely low (16%). In a study investigating the temporal patterns of meeting the screen time guideline among the same population at an earlier age, a decrease of 26 percentage points in adherence rate of children at age 54-months (18.4%) was observed compared to 24-months (44.4%) [45]. Collectively, this decreasing trend in screen time adherence among New Zealand children warrants immediate attention considering the detrimental health impacts associated with high screen time [2]. Consistent with other studies [46, 47], children with mothers who have higher educational qualifications had a greater adherence rate to screen time guideline. Additionally, children’s screen time was significantly associated with child’s ethnicity with European were more likely to meet the screen time guideline compared to other ethnicities. Similar observations were made in other studies in New Zealand [45] and other countries [48] where minoritised ethnic groups were more likely to exceed the screen time recommendations. We also found that those residing in rural areas had higher odds of meeting the screen time guideline. Others have also found that rural children tend to have less screen time compared to their urban peers [11]. This observation might mean that children in rural settings have higher opportunities to spend time in outdoor activities and therefore are less engaged in screen-based activities compared with urbans children.

In this study, 62.5% of the children met the sleep duration recommendation, which is less than Australian and UK children [9], but higher than American, Canadian, Chinese, African [9], and Chilean children [49]. Only a small proportion of children (10.6%) met the combined 24-h Movement Guidelines. This observation of low adherence to these guidelines among children is in agreement with previous evidence from several countries showing that only 5–15% of children aged 9–11 met all three recommendations in the 24-h Movement Guidelines [9, 49, 50].

This study is one of only a small number to investigate the sociodemographic factors of meeting the 24-h Movement Guidelines. In our study, meeting the combined 24-h Movement Guidelines was associated with the child’s ethnicity and mother’s education. As shown in a recent review [5], a limited number of studies have examined sociodemographic correlates of meeting the combined 24-h Movement Guidelines among children [10, 11]. These studies suggest that there is an association between parental education, outdoor time, school location (urban vs rural), maternal activity level and TV viewing time before pregnancy and meeting the combined 24-h Movement Guidelines [10, 11]. Clearly, more studies need to investigate the sociodemographic correlates of meeting these guidelines to provide evidence for developing more effective interventions targeting those who are more likely to engage in unhealthy time-use patterns.

There are several strengths to this study. We used 24-h accelerometery to measure 24 time-use behaviours, and the 24-h time-use compositions of children were described from two perspectives: activity intensity (using accelerometer-derived counts) and activity type (using machine learning algorithms). Additionally, we applied CoDA to investigate sociodemographic differences in 24-h time use behaviours. To our knowledge, this is the first study in which CoDA methods have been applied to determine the group differences in time-use compositions among children. Adequately accounting for the compositional nature of time-use behaviours, these methods should be used while dealing with compositional data [21]. There are also several limitations which need to be considered. Firstly, there were significant differences between sociodemographic characteristics of those included and excluded in this study, which could reduce the generalisability of the findings. Also, screen time was parent-reported which is prone to bias [51], and 25% of the children were missing screen time data which may limit the representativeness of the screen time results. Additionally, as children with at least 1 day of valid accelerometer data were included in the analysis, potential variability between weekday and weekend time-use patterns, and individual vs. multiple days, were not taken into account. Finally, the cross-sectional nature of the study precludes any causative conclusions to be drawn regarding the sociodemographic correlates of 24-h time-use patterns.

## Conclusions

In this study, child gender, ethnicity, household income, and household deprivation were associated with the 24-h activity intensity and activity type compositions in New Zealand children. Girls were more at risk of lower MVPA (and walking and running) compared to boys. Asian children had higher LPA, but less sleeping time compared to the other ethnicity groups. Children from high deprived households were at higher risk of spending more time sitting and less time walking or running compared to the children from less deprived households. Overall, a small proportion of New Zealand school-aged children met the combined 24-h Movement Guidelines. Sociodemographic factors, including child gender, ethnicity, mother’s education, and household area were associated with meeting these Guidelines. These findings may help to design more effective future interventions to promote optimal 24-h movement patterns for New Zealand children.

## Supplementary Information

**Additional file 1: Table S1.** Variation array of the four-part activity intensity composition. **Table S2.** Variation array of the five-part activity type composition.

**Additional file 2: Supplemental Figure S1.** Ternary plots showing the difference between gender, for activity intensity (top) and activity type (bottom). The small points represent individual participants, the large points and crosshairs represent the compositional means for each group, while the polygon indicates the 95% confidence ellipse. The axis units are proportions (%) of time. **Supplemental Figure S2.** Ternary plots showing the difference among ethnicities, for activity intensity (top) and activity type (bottom). The small points represent individual participants, the large points and crosshairs represent the compositional means for each group, while the polygon indicates the 95% confidence ellipse. The axis units are proportions (%) of time. **Supplemental Figure S3.** Ternary plots showing the difference among household income groups, for activity intensity (top) and activity type (bottom). The small points represent individual participants, the large points and crosshairs represent the compositional means for each group, while the polygon indicates the 95% confidence ellipse. The axis units are proportions (%) of time.

## Data Availability

The data contain personal information and are not publicly available but can be requested. A guide for researchers and policy-makers who are interested in using the *Growing Up in New Zealand* datasets is available at https://www.growingup.co.nz/using-data
