# Supplementary material for: Sociodemographic differences in 24-hour time-use behaviours in New Zealand children
Source: Int J Behav Nutr Phys Act. 2022 Oct 4;19:131. doi: 10.1186/s12966-022-01358-1 (PMC9531491; doi:10.1186/s12966-022-01358-1)
Supplement: Supplementary file 1 — Additional file 1: Table S1. Variation array of the four-part activity intensity composition. Table S2. Variation array of the five-part activity type composition. [file 12966_2022_1358_MOESM1_ESM.docx]

Table S1 presents the variation array of the activity intensity composition. The upper triangle of the variation array is the variation matrix. This describes the pairwise variation between parts and is loosely equivalent to the univariate standard deviation. The highest variation is observed in the log ratio of MVPA vs. sedentary. This suggests that as MVPA increases, sedentary tends to decrease to compensate (and vice versa). The lower triangle of the variation array presents the mean of the pairwise log ratios. The positive numbers mean the log-ratio denominators are, on average, smaller than the numerators. For example, the average ln(Sedentary/MVPA) is 1.52, meaning that the duration of sitting is greater than the duration of walking. To calculate how much more, the log-ratio can be exponentiated, exp(1.72) = 5.58, meaning the ratio of sitting to walking is approximately 5.6 to 1.

**Table S1**. Variation array of the four-part activity intensity composition.

|  |  | **Mean Variation of the Pairwise Logratio** | | | | |
| --- | --- | --- | --- | --- | --- | --- |
|  |  | **Sedentary** | **Light** | **MVPA** | **Sleep** |  |
| Denominator of  logratio | Sedentary | . | 0.08 | 0.19 | 0.04 |  |
|  | Light | 0.33 | . | 0.10 | 0.05 |  |
|  | MVPA | 1.52 | 1.19 | . | 0.13 |  |
|  | Sleep | -0.25 | -0.58 | -1.77 | . |  |
|  |  | Mean of the pairwise logratio | | | | |

The upper triangle is the variation of the logratios (i.e., variation matrix). The lower triangle is the mean of the logratio.

**Table S2**. Variation array of the five-part activity type composition.

|  |  | **Mean Variation of the Pairwise Logratio** | | | | |
| --- | --- | --- | --- | --- | --- | --- |
|  |  | **Sitting** | **Standing** | **Walking** | **Running** | **Lying** |
| Denominator of  logratio | Sitting | . | 0.24 | 0.22 | 0.88 | 0.08 |
|  | Standing | 1.14 | . | 0.15 | 0.89 | 0.18 |
|  | Walking | 1.53 | 0.38 | . | 0.58 | 0.15 |
|  | Running | 4.26 | 3.12 | 2.74 | . | 0.82 |
|  | Lying | -0.37 | -1.51 | -1.89 | -4.63 | . |
|  |  | Mean of the pairwise logratio | | | | |

The upper triangle is the variation of the logratios (i.e., variation matrix). The lower triangle is the mean of the logratio.
