# Supplementary material for: Sociodemographic differences in 24-hour time-use behaviours in New Zealand children
Source: Int J Behav Nutr Phys Act. 2022 Oct 4;19:131. doi: 10.1186/s12966-022-01358-1 (PMC9531491; doi:10.1186/s12966-022-01358-1)
Supplement: Supplementary file 2 — Additional file 2: Supplemental Figure S1. Ternary plots showing the difference between gender, for activity intensity (top) and activity type (bottom). The small points represent individual participants, the large points and crosshairs represent the compositional means for each group, while the polygon indicates the 95% confidence ellipse. The axis units are proportions (%) of time. Supplemental Figure S2. Ternary plots showing the difference among ethnicities, for activity intensity (top) and activity type (bottom). The small points represent individual participants, the large points and crosshairs represent the compositional means for each group, while the polygon indicates the 95% confidence ellipse. The axis units are proportions (%) of time. Supplemental Figure S3. Ternary plots showing the difference among household income groups, for activity intensity (top) and activity type (bottom). The small points represent individual participants, the large points and crosshairs represent the compositional means for each group, while the polygon indicates the 95% confidence ellipse. The axis units are proportions (%) of time. [file 12966_2022_1358_MOESM2_ESM.docx]

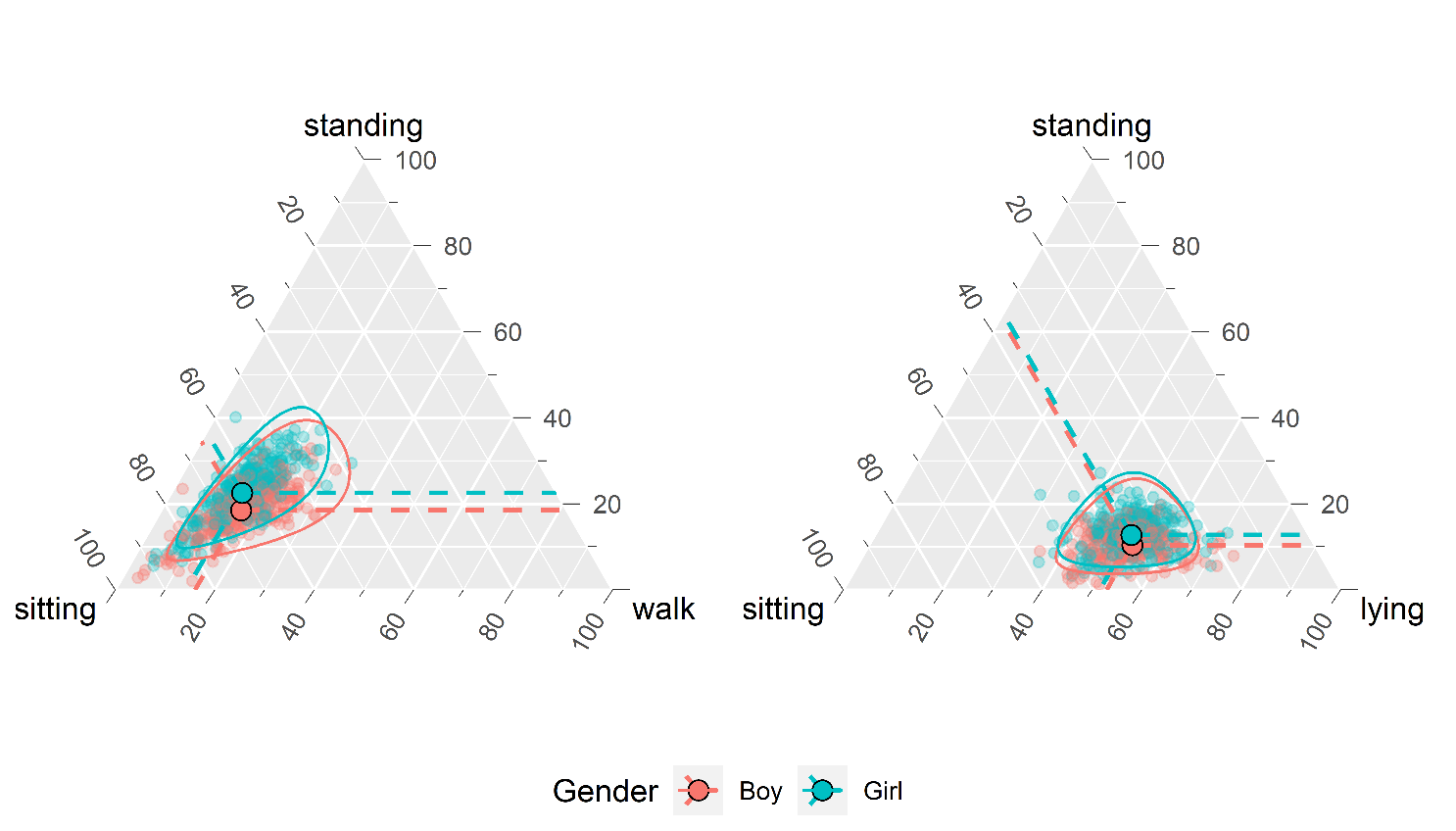

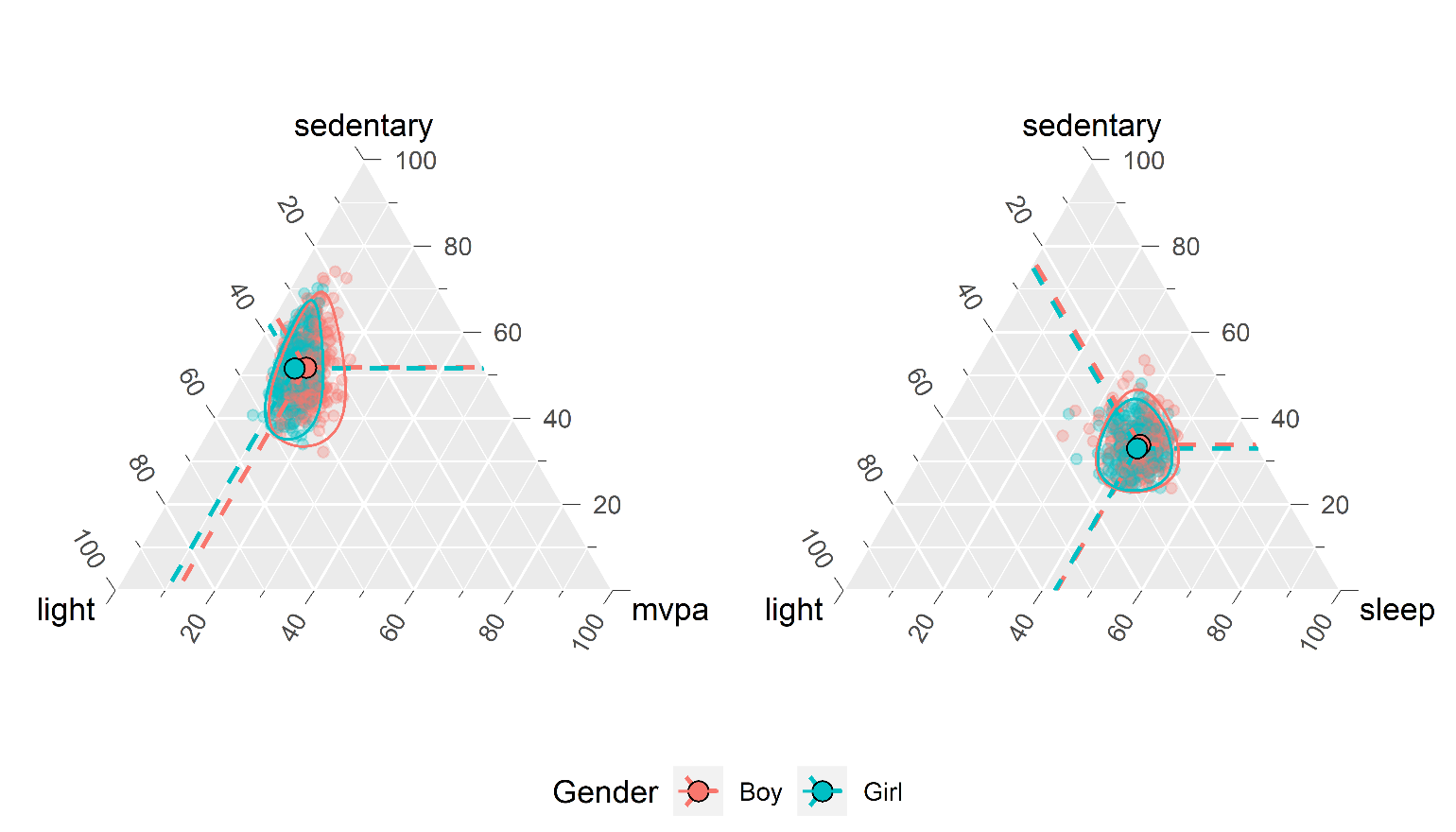


**Supplemental Figure S1.** Ternary plots showing the difference between gender, for activity intensity (top) and activity type (bottom). The small points represent individual participants, the large points and crosshairs represent the compositional means for each group, while the polygon indicates the 95% confidence ellipse. The axis units are proportions (%) of time.


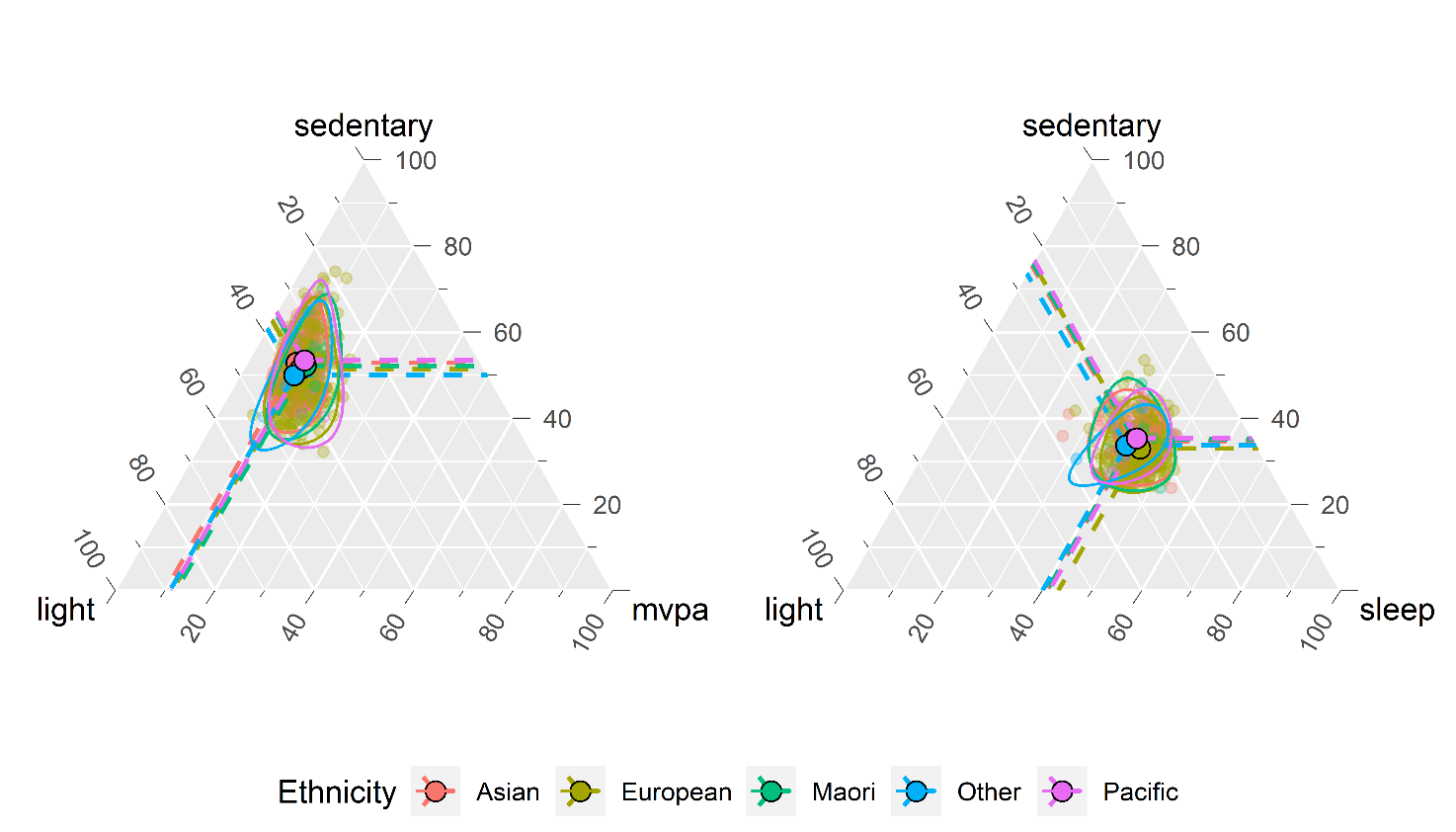

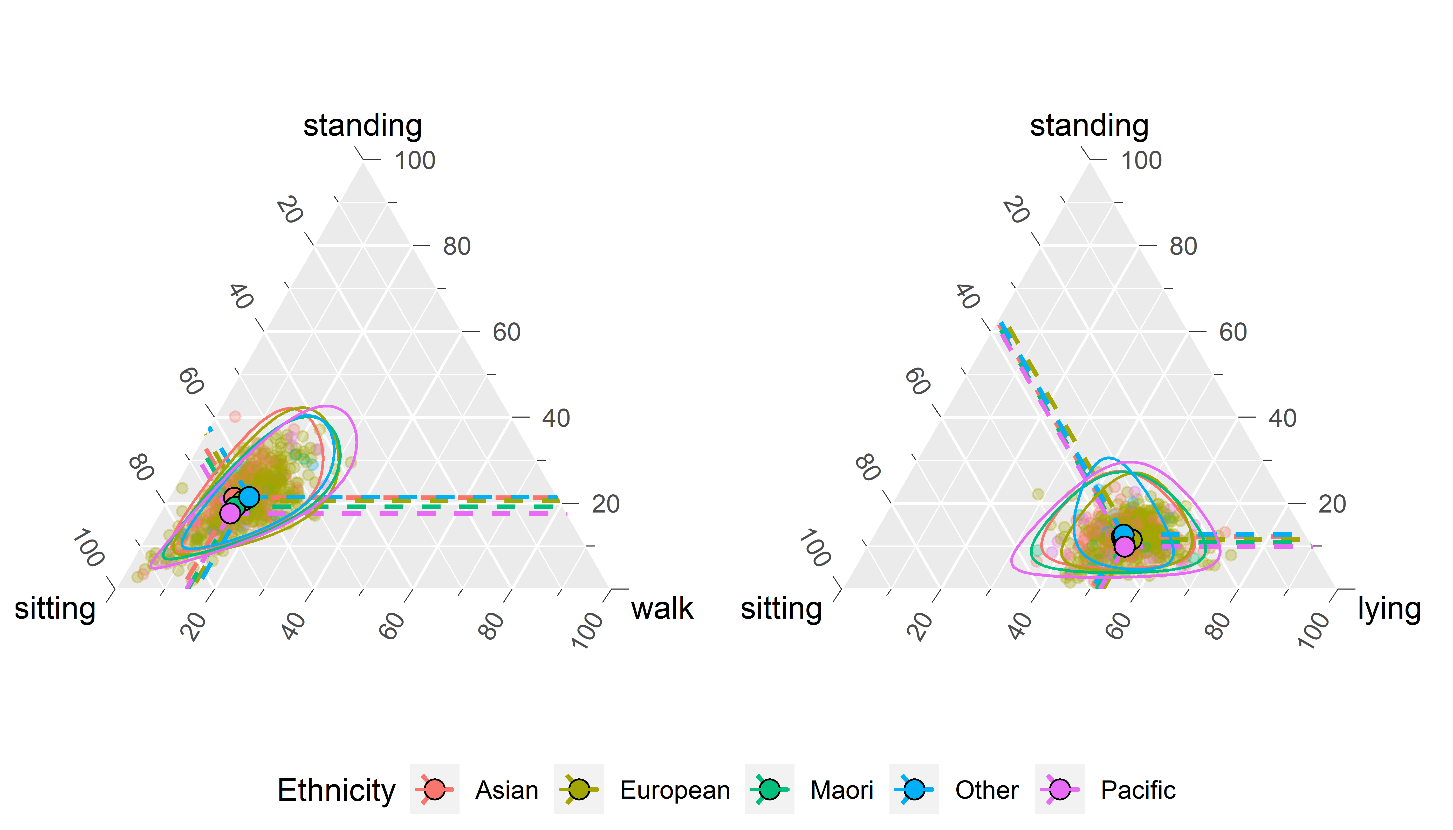


**Supplemental Figure S2.** Ternary plots showing the difference among ethnicities, for activity intensity (top) and activity type (bottom). The small points represent individual participants, the large points and crosshairs represent the compositional means for each group, while the polygon indicates the 95% confidence ellipse. The axis units are proportions (%) of time.


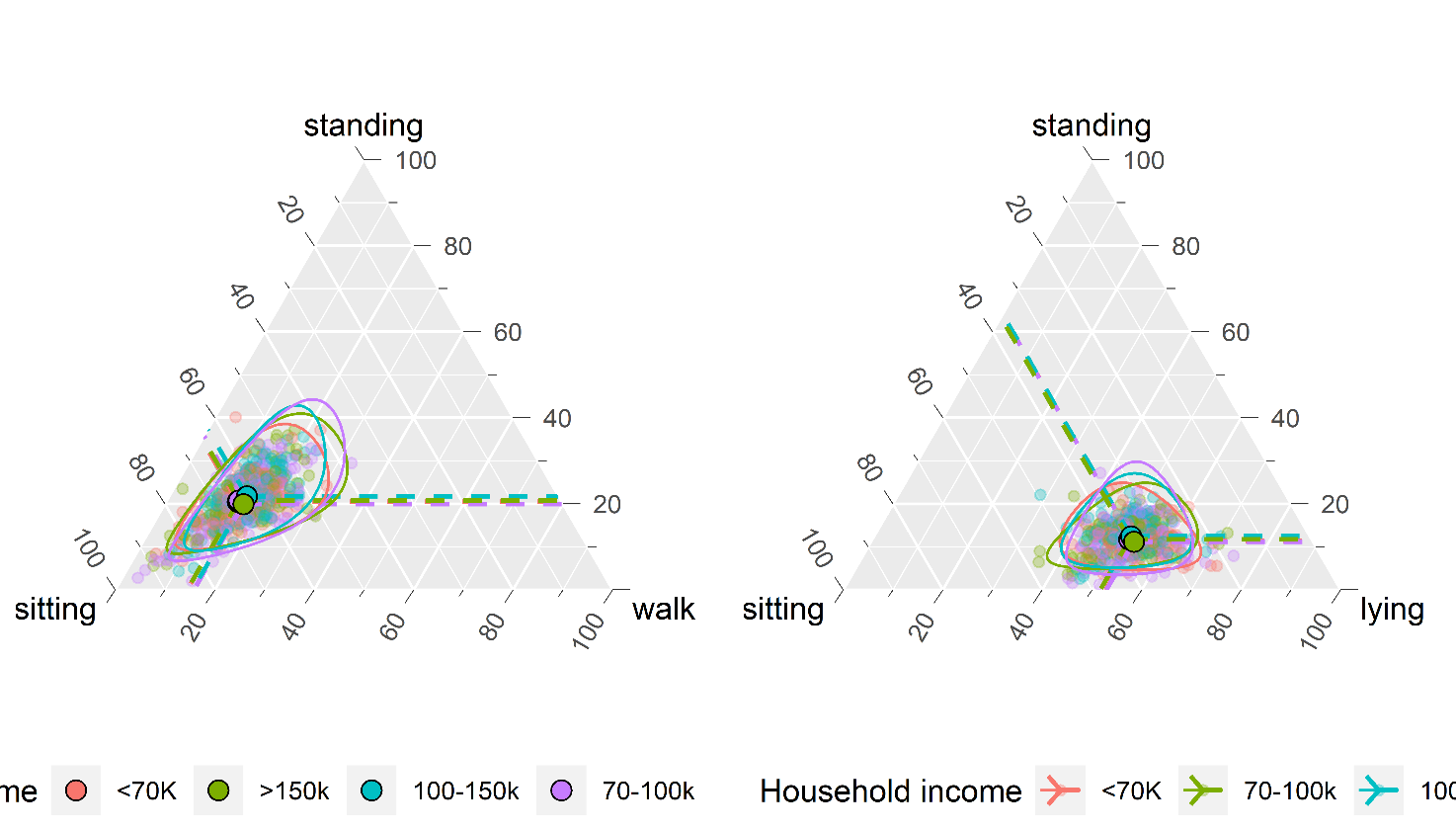

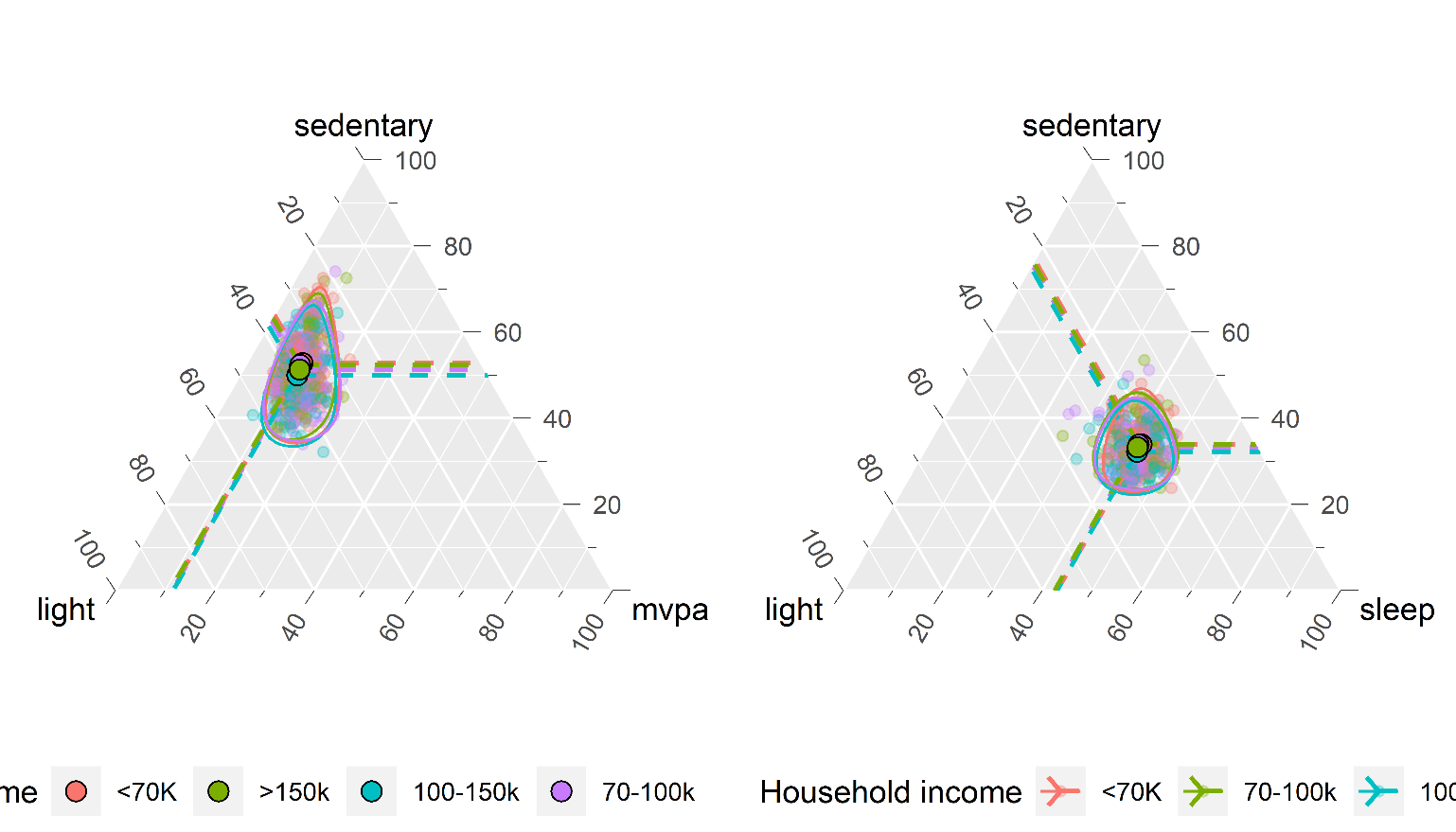

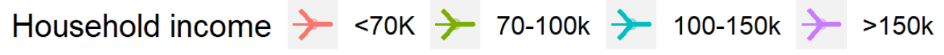


**Supplemental Figure S3.** Ternary plots showing the difference among household income groups, for activity intensity (top) and activity type (bottom). The small points represent individual participants, the large points and crosshairs represent the compositional means for each group, while the polygon indicates the 95% confidence ellipse. The axis units are proportions (%) of time.
